# Editorial: Cognitive, Psychological, and Psychiatric Consequences of the Coronavirus (COVID-19) Pandemic in the Population of Older Persons With Cognitive Impairment, Dementia, and/or Neuropsychiatric Disorders

**DOI:** 10.3389/fpsyt.2021.748963

**Published:** 2021-10-14

**Authors:** Katie Palmer, Miia Kivipelto, Walter Gianni, Nerisa Banaj, Gianfranco Spalletta

**Affiliations:** ^1^Division of Clinical Geriatrics, Department of Neurobiology, Care Sciences and Society, Karolinska Institutet, Solna, Sweden; ^2^FINGERS Brain Health Institute, Stockholm, Sweden; ^3^Medical Unit Aging, Karolinska University Hospital, Solna, Sweden; ^4^Ageing Epidemiology (AGE) Research Unit, School of Public Health, Imperial College London, London, United Kingdom; ^5^Institute of Public Health and Clinical Nutrition and Institute of Clinical Medicine, Neurology, University of Eastern Finland, Kuopio, Finland; ^6^Department of Internal Medicine and Geriatric Medicine, University Hospital Policlinico Umberto I, Rome, Italy; ^7^Laboratory of Neuropsychiatry, IRCCS Santa Lucia Foundation, Rome, Italy

**Keywords:** COVID-19, psychology, psychiatry, elderly, cognitive impairment (CI), dementia, neuropsychiatry, psychopathology

## Introduction

The COVID-19 pandemic has created an unprecedented crisis throughout the world, which has led to emergency measures to control the rates of SARS-CoV-2 infection and a relocation of healthcare resources to monitor, diagnose, and treat COVID-19. Although there has been an understandable concern that older individuals, particularly men and those with preexisting comorbidities, have a higher risk of COVID-19 disease complications and mortality (1), older individuals have also faced wider risks related to their long-term health and well-being in relation to public health measures that were initiated at the start of the pandemic to control infection rates.

During 2020, in the first wave of the pandemic, most countries devised public health measures that had the primary aim to decrease rates of infection and reduce the burden of COVID-19 on already stretched healthcare systems, including social distancing, lockdowns, quarantines, and reduction of non-urgent medical services, among others. Although there was an understandable urgency to control the pandemic, the short- and long-term risks of these measures were unknown and there was concern that older persons, especially those with noncommunicable diseases such as dementia disorders, may be at particularly high risk of negative outcomes associated with these measures, particularly psychological effects, psychiatric symptoms, and declining health due to reduced access to healthcare.

It is well established that prevention can be useful for reducing cognitive deterioration, both primary (to prevent cognitive impairment) and secondary prevention (to slow down ongoing cognitive decline). Guidelines on dementia prevention by the World Health Organization (2) and Lancet Commission (3, 4) describe 12 modifiable risk factors, including physical activity, appropriate nutrition, social support and social interactions, and cognitive stimulation as important measures for reducing cognitive decline. During the current COVID-19 crisis, where many countries have been using isolation and lockdown procedures, it is likely that these protective factors are compromised, particularly in older persons with or without mental disorders and those at risk of developing dementia, which may cause a cascade of events leading to cognitive impairment.

In this Research Topic, we aimed to investigate how the COVID-19 pandemic has affected older people, especially those with cognitive impairment, from a range of perspectives to help establish factors associated with poor physical, cognitive, and mental health. One important focus of the current Research Topic was to examine how the pandemic has affected older individuals in different living situations, including those who live alone, residents in Long-Term Care Facilities (LTCF), and those receiving support from family and other informal caregivers. It is important to note that the studies in this Research Topic mostly collected data during 2020, mainly during the first wave of COVID-19. Therefore, the insights relate only to this time period. The Research Topic is comprised of 44 contributions on a wide range of themes that address how the pandemic has affected the lives of older people from multiple perspectives, including 29 original articles, 5 reviews, 9 opinion and perspective articles, and a study protocol (Porcari et al.), as summarized below.

## Psychological and Neurological Effects in Patients With COVID-19

Several papers highlighted the psychological effects of COVID-19 in both the acute phase of the disease and post-infection. A scoping review of 85 articles published between 2019 and May 19th 2020 (Wenting et al.) described that the neurological manifestations of COVID-19 vary from mild (e.g., loss of taste and smell, dizziness, headache) to severe (e.g., ischemic stroke, encephalitis). The authors suggested that underlying mechanisms of central nervous system (CNS) involvement could be both direct (neurotropic) and indirect (as a result of thrombotic complication, inflammatory consequences, hypoxia, blood pressure dysregulation). In their hypothesis and theory article, Panariello et al. proposed possible mechanisms underlying neuropsychiatric manifestations in COVID-19 that appear to develop in patients with and without pre-existing neurological disorders. In a sample of older COVID-19 patients with psychosis in North Italy, Rozzini et al. reported that delirium, particularly the hypokinetic form, is related to a high risk of mortality in patients with COVID-19, especially in the presence of dementia. In the observed patients, 43% exhibited hypokinetic delirium with lethargy and confusion, of whom half died.

## The Effects of the COVID-19 Pandemic on the General Population and Healthy Older People

Several studies in the Research Topic focused on identifying how lifestyle and health-related behaviors of older individuals changed during the pandemic, with a mixture of both positive and negative changes. These results are important, since many of these are risk factors for cognitive decline and are modifiable (Lehtisalo et al.), such as smoking, obesity, depression, physical inactivity, infrequent social contact, and excessive alcohol consumption (4).

Ongoing epidemiological studies with pre-pandemic measures have provided useful insights into intra-individual changes that have occurred in older persons as a result of the pandemic. In a population-based study in the Czech Republic, involving participants from the Kardiovize study (5),Novotný et al. observed increased stress levels and more severe depressive symptoms in participants during the COVID-19 lockdown compared to baseline levels measured before the lockdown. This increase in mental distress was more severe in women and was associated with illness perception, personality characteristics such as feelings of loneliness, and several lifestyle components (e.g., nutrition, sleep quality, exercise etc.). Individuals who perceived COVID-19 as emotionally threatening exhibited the highest increase in stress levels and severity of depressive symptoms. Although this increase in mental distress was present in all ages, cross-sectionally the older age group showed the lowest levels of mental distress prior to and during the lockdown. Several cross-sectional surveys reported similar symptoms in the general population during the first wave of the pandemic. In Greece, Parlapani et al. found that a large percentage of individuals reported moderate to severe depressive (81.6%) and anxiety (84.5%) symptoms, as well as sleep disturbances (37.9%) and suicidal ideation (37.9%) during the first wave of the COVID-19 pandemic. The analyses highlighted that women had a significantly higher level of COVID-19 related fear, severe depressive symptoms, sleep disturbances, and higher levels of intolerance of uncertainty. Moreover, participants living alone showed higher level of loneliness; intolerance of uncertainty was found as a predictor of sense of loneliness. Similarly, in the UK (Robb et al.) a survey revealed that 12.8% of cognitively healthy older adults reported increased depressive symptoms and 12.3% had increased anxiety. These symptoms were higher in women, younger participants, people who were single, widowed, or divorced, as well as those who were living alone. Further, individuals who reported having little sleep and expressed feelings of loneliness were more likely to feel more depression and anxiety symptoms.

A Spanish study on persons aged 60 and over (Rodríguez-González et al.) reported that although more than two thirds of participants had an open space at home, 65.7% did less physical activity and only one third continued doing activities to promote healthy aging. There was a higher presence of negative feelings during quarantine in participants who did not have open spaces at home. The authors also observed that greater scores on resilience were negatively correlated with age and negative feelings, and positively correlated with the size of the social network and positive feelings.

An Italian study on community-dwelling people at increased risk of dementia (e.g., subjective cognitive decline and MCI) (Di Santo et al.) reported negative lifestyle changes that are potentially harmful to future cognitive decline. More than one third reduced their physical activity during the pandemic and nearly 70% reported an increase in idle time. Individuals also reported a decrease in adherence to the Mediterranean diet and more than a third reported weight gain. About one fifth were depressed, and this was significantly associated with living alone or having a poor relationship with cohabitants, low sleep quality, and not owing a pet. More than a quarter (27.2%) reported that they had often felt sad, depressed, or downcast so much since the start of the lockdown that nothing could cheer them up. Community-dwelling people at increased risk of cognitive decline were also the focus of a Finnish population-based survey (Lehtisalo et al.), where a mixture of positive and negative lifestyle changes were observed during the pandemic. Although about one third reported a decrease in physical activity a large proportion of people were able to keep up healthy eating habits, with many increasing their vegetable and fruit consumption. Self-rated health and quality of life generally remained stable, but 21% reported more feelings of loneliness and 15% felt that their memory had been getting worse during the pandemic. Older people and those living alone seemed more susceptible to loneliness and negative changes. In contrast, Bidzan-Bluma et al. found that older people (aged 60+) in a Polish and German population study rated their quality of life, life satisfaction, sleep quality, and well-being during the pandemic higher than younger people. Further, they experienced lower levels of trait anxiety. However, the authors noted that the older participants were generally financially stable and had high education (>60% with university education), which may have influenced the results. Similarly, Rossi et al. reported that age moderates the mediating effect of resilience in the relationship between COVID-19-related stressful events and depressive and anxiety symptoms and perceived stress in an Italian sample. Older adults (age 60+) reported lower levels of depressive symptoms, anxiety, and stress than younger persons, and had higher levels of resilience. The authors suggested that resilience in older adults is less influenced by stressful events, and this could be one of the reasons accounting for the better mental health outcomes observed in the older age group during the pandemic.

After performing a search of the existing literature, Fontes et al. proposed some intervention and preventive measures to mitigate and reduce the risk of psychological and psychiatric disorders in older persons. They proposed expanding telehealth services for older people and their relatives (for answering questions about psychological and psychiatric symptoms and establishing contact to monitor and access medication and non-pharmacological adjuvant therapy) and using telepsychiatry as a screening and assessment tool. They also emphasized the needed to prepare training materials for healthcare on good mental health practices during the pandemic and to offer educational materials for individuals to increase awareness of interacting and caring for older relatives.

In a perspective paper, Lozupone et al. reinforced the importance of correct assessment of social frailty in terms of the prevention of late-life neuropsychiatric disorders, particularly in the COVID-19 era. One study also examined how COVID-19 affected patients after recovery; Janiri, Kotzalidis, et al. reported a higher frequency of psychological distress in patients aged over 60 after the acute phase of illness, which in turn may be associated with impaired emotional regulation and higher scores on depressive and cyclothymic temperaments. A literature review (Manca et al.) reported evidence from 8 papers showing that different neuropsychiatric symptoms emerged and/or worsened in older adults with and without dementia as a consequence of COVID-19 infection. Further, a study by Banerjee and Rao conducted in India on older individuals with transgender identity, revealed that they were at increased emotional and social risks during the COVID-19 pandemic, particularly marginalization, the dual burden of “age” and “gender,” and multi-faceted survival threats (physical, emotional, financial). Social rituals, spirituality, hope, and acceptance of “gender dissonance” emerged as the main coping factors.

## Residents in Long-Term Care Facilities During the Pandemic

de Girolamo et al. reported higher mortality rates in LTCF residents in Northern Italy when compared to expected values of mortality rates among the older general population living in the community; mortality increased about four times during the pandemic when compared to previous years. Other adverse events were also seen during the pandemic in these settings; Lombardo et al. found that one third of LTCFs participating in their study reported at least one adverse event (defined as any harm or injury resulting from medical care or the failure to provide care), during March 24 to May 5 2020. Several factors were associated with the occurrence of adverse events in these facilities, including having a higher bed capacity (more than the median of 60 beds), increased use of psychiatric drugs, physical restraint measures, residents hospitalized due to flu-like symptoms, and being located in specific geographic areas (i.e., North-West, North-East Italy). The pandemic was also shown to affect visitors to LTCFs; an online survey in Ireland (O'Caoimh et al.) reported that many LTCF visitors experienced poor psychological and emotional well-being during the COVID-19 lockdown. Further, visitors of residents with cognitive impairment showed significantly lower well-being than those without.

## The Effect of COVID-19 Lockdowns and Quarantine on Patients With Mild Cognitive Impairment (MCI) and Dementia Disorders

Many articles in this Research Topic highlighted that the pandemic affected individuals with dementia disorders, MCI, and other conditions, particularly with regard to behavioral and neuropsychiatric symptoms. In a systematic review, Simonetti et al. observed that neuropsychiatric symptoms of dementia (especially apathy, anxiety, and agitation) during COVID-19 appear to arise from pandemic-related social restrictions, while Manca's et al. review highlighted that that delirium, agitation, and apathy were the symptoms most commonly detected in older adults during the pandemic, especially in people with dementia. An Italian multisite study in 87 memory clinics (Cagnin et al.) reported a rapid increase of Behavioral and Psychological Symptoms of Dementia (BPSDs) in ~60% of dementia patients. The pattern of BPSDs varied according to dementia type, disease severity, and sex; anxiety and depression were associated with Alzheimer's Disease, mild to moderate dementia severity, and being female, whereas patients with dementia with Lewy bodies had a significantly higher risk of worsening hallucinations and sleep disorders. Frontotemporal dementia was associated with wandering and appetite changes. Overall, irritability, apathy, agitation, and anxiety were the symptoms that were most frequently reported to worsen during the pandemic, while sleep disorders and irritability were the most reported new symptoms. Similar behavioral symptoms were observed in community-dwelling patients with dementia in an Argentinian study; Cohen et al. reported increased anxiety, insomnia, depression, and a worsening of gait disturbances during the pandemic in these patients. Anxiety, depression, and insomnia were more common in individuals with mild compared to severe dementia. Family members also reported an increased use of psychotropic drugs to control behavioral symptoms in the dementia patients (specifically a 20% increase for antipsychotics, 15% for benzodiazepines, 6% for hypnotics, and 10% for antidepressants).

A study conducted in an Alzheimer Center in the Netherlands (van Maurik et al.) showed that 44% of patients with cognitive impairment were concerned about faster cognitive decline. Both patients with symptomatic cognitive impairment and cognitively normal patients (i.e., with subjective cognitive decline) reported an increase of one or more psychological symptoms as a result of the pandemic-related measures. Caregivers reported an increase in patients' apathy (54%), a change in sleeping behavior (48%), increased repetitive behavior (34%), and patient aggression (30%). Social isolation and reporting one or more psychological symptoms were determinants for worries for faster cognitive decline.

A Japanese study provided important insight into the situation faced by patients with dementia or MCI who live alone (Hashimoto et al.). Most patients who lived alone did not limit their outings or activities during the COVID-19 outbreak, whereas more than half of the patients who lived with their families reduced their frequency of going out. The researchers used an original questionnaire to caregivers and/or patients to evaluate how the patient's current state compared to the prepandemic period. When asked “Did the COVID-19 outbreak increase the patients' mental stress?,” patients with dementia or MCI reported significantly less mental stress than caregivers, regardless of living conditions.

## Patients With Parkinson'S Disease, Down Syndrome, and Tumors

An Italian study (Baschi et al.) on patients with PD, MCI, or both (PD-MCI), showed a worsening of cognitive, behavioral (both pre-existing and new), and motor symptoms during the COVID-19 lockdown, particularly those with PD and MCI. Compared to PD patients without cognitive impairment, PD-MCI were more like to decline in Instrumental Activities of Daily Living functions. Further, they had higher frequencies of all NPI symptoms except appetite/eating disturbances and a significantly higher frequency of cognitive impairment, fatigue, and speech problems. These symptoms resulted in an increased caregiver burden in about a quarter of cases. Similarly, Janiri, Petracca, et al. reported that a quarter of PD outpatients with lifetime psychiatric symptoms showed a worsening of psychiatric symptomatology during the COVID-19 outbreak, especially depression and insomnia. Lifetime pre-existing delusions, having received antipsychotics, and not having received mood stabilizers were associated with subjective worsening of psychiatric symptomatology due to the COVID-19 pandemic.

Villani et al. investigated Italian adults with Down Syndrome using an analysis comparing pre- and post-lockdown evaluations. After the lockdown period there was a significant worsening in social withdrawal, instrumental activities of daily living, and depression together with a significant improvement in aggressive behavior. Büssing et al. reported lower well-being among tumor patients living in Germany, especially in the younger population. More than half were worried about being infected and having a complicated disease course. Patients noticed changes in their attitudes and behaviors because of the pandemic-related restrictions, including worrying reflections and loneliness, interest in spirituality, and intense relationships.

## Caregiver Burden During the Pandemic

Early in the pandemic, there was a significant disruption to healthcare and formal care services due to the potential risk of SARS-CoV-2 infection in staff and patients, as well as a redistribution of healthcare budgets to focus on COVID-19 diagnosis, prevention, and treatment. Consequently, informal caregivers, particularly those of patients with dementia and other neurocognitive disorders, were often relied on to counterbalance the reduction of formal services, which may negatively affect their health and well-being. In a Dutch study on pre-dementia memory clinic patients (van Maurik et al.), care was discontinued during the COVID-19 pandemic for three quarters of symptomatic patients, and this was strongly associated with caregiver burden. More than half of caregivers reported a higher caregiver burden, which was also associated with psychological and behavioral problems, and almost one third reported a need for more support. An Italian multisite study (Cagnin et al.) found stress-related symptoms in two-thirds of dementia patient caregivers during COVID-19 and, in China, Li et al. reported a high prevalence of anxiety and depressive symptoms among caregivers. Being female was an independent risk factor for experiencing anxiety symptoms while pre-existing mental disorders increased the risk of depressive symptoms. In Brazil (Penteado et al.), a study on patients with neurocognitive disorders and Down syndrome reported that clinically relevant neuropsychiatric symptoms had a significant impact on caregiver distress during the COVID-19 pandemic. Apathy, aberrant motor behavior, sleep disorders, and psychoses contributed most to an increase in caregiver burden. Interestingly, interventions may help to reduce the risk of caregiver burden, as reported by a study in Northern Italy (Cravello et al.) on patients with dementia or cognitive decline whose related caregivers had attended a structured family support course before the COVID-19 lockdown. After lockdown, the patients did not have a worsening of neuropsychiatric symptoms and, although their functional abilities declined, their caregivers experienced a decrease in caregiver burden in comparison to the pre-lockdown period. This provides promising insight into how comprehensive family support interventions that teach, train, and aid caregivers of patients with cognitive disorders can reduce caregiver burden even in negative periods such as the COVID-19 pandemic.

## Effects of the COVID-19 Pandemic on Clinical Activities and Healthcare

During the first year of the pandemic, there was a rapid change in routine clinical activities for non-urgent medical conditions, due to public health restrictions and the potential risk of SARS-CoV-2 infection in both patients and healthcare professionals. In the first wave, cancellations in dental healthcare (43%), home aid (30%), and rehabilitative services (53%) were reported in a Finnish population-based survey of older persons at risk of cognitive decline. Cohen et al. reported that rehabilitation services had been discontinued due to the lockdown in most community-dwelling dementia patients in their Argentinian study. Further, Spalletta et al. reported a substantial decrease in scheduled appointments in an Italian Memory Clinic in March–April 2020 compared to the same period in 2019 due to the Government's restrictive measures. They estimated that many patients with dementia and cognitive disorders missed crucial appointments (66.7% of patients who were due to have first appointments and 77.4% with follow-up appointments), resulting in a delay in initial diagnosis and initiation of treatment. Korsnes et al. described that most of the patients at the Department of Old Age Psychiatric 24-h unit in Norway welcomed the strict measures that were applied in the clinic (including a visitation ban for inpatients and a reduction in outpatient consultations). Interestingly, many individuals reported that they were not very scared of getting COVID-19 and many did not believe that they would die if they were infected. On the other hand, employees were concerned about how the COVID-19 crisis would influence their health and well-being at work.

In a perspectives article, D'Cruz and Banerjee expressed concerns regarding the care of persons living with dementia in India, discussing that they face dual risks due to both age and cognitive decline, which are accentuated by the pandemic. The authors suggested that pandemic control in India can be best achieved when persons living with dementia are made part of, and advocates for, care rather than mere recipients. Through interviews with dementia care physicians in Southern India, Banerjee et al. outlined the major concerns and barriers to care of persons with dementia during the pandemic. Although an overarching theme was that telemedicine is the future of dementia care in India most participants perceived ambiguity related to newly-released national telepsychiatry guidelines.

## Temporary Care Facilities, Remote Assessment, and Digital Solutions for Healthcare During and After the Pandemic

COVID-19 heightened the need for remote assessment of older people, especially as they have a higher risk of COVID-19 complications and thus, have often been encouraged to adhere strictly to social distancing measures. Owens, Hindus et al. provided recommendations from a Patient Advisory board of a European project that included a set of prioritized functional domains sensitive to the early stages of Alzheimer's Disease and a set of remote measurement technologies capable of targeting them. A review of the existing literature (Owens, Ballard, et al.) highlighted several challenges for remote memory clinics related to internet access, computer skills, limited evidence base, and regulatory and data protection issues. The authors suggested that digital biomarkers collected remotely may have significant potential for diagnosis and symptom management in older adults and proposed a framework and pathway for how technologies can be implemented to support remote memory clinics. Sousa Alves et al. conducted a systematic review of pre-pandemic home-setting psychoeducation interventions for behavioral changes in dementia, to identify potential solutions for the COVID-19 era. They observed that most of the psychosocial and psychoeducational interventions described were person-centered strategies based on the cognitive-behavioral approach or informational tools to enhance care providers' knowledge of dementia. Most studies achieved successful results in handling BPSD and mood-anxiety symptoms of care providers, contributing to an overall improvement in dyad life quality. They concluded that low-cost techniques, tailored to the dyad well-being, with increasing use of technology through friendly online platforms and application robots, can be an alternative to conventional assistance during COVID-19 Pandemic.

Debas et al. reported their experience from a temporary care facility for institutionalized patients with major neurocognitive disorder and BPSDs during the SARS-CoV-2 pandemic in Canada. Due to their expertise as a multidisciplinary team specialized in BPSD management, they were asked to support staff in the temporary care facility who had little experience in dementia care. This had a positive impact on non-professionals' sense of effectiveness in addressing patients' neuropsychiatric symptoms.

Keng et al. provided recommendations on how to address challenges faced by individuals with BPSD and their caregivers during the pandemic with a proactive approach: implementing infection control strategies, monitoring the long-term biological and psychosocial effects of COVID-19 in patients with BPSDs, using evidence-based structured psychosocial and biological interventions through innovative means such as virtual and individualized care to manage BPSD, use of structured and algorithmic models of care, and appropriate use of psychosocial interventions across healthcare settings.

Soares et al. gave recommendations on telemedicine as an important alternative method of assistance for BPSD management. They discussed how telemedicine can expand access to clinical resources and link healthcare providers with patients and their caregivers, thereby overcoming the reductions in face-to-face appointments and providing a balance between the need for both social distancing and specialized consultation. They also described how it can help caregivers by providing guidance on non-pharmacological measures to control symptoms that are adapted to the new social distancing and lockdown scenarios.

Although many articles discussed the benefits of digital medicine tools, Martins Van Jaarsveld importantly discussed the increased negative effects that the digital divide is having in the older population during the COVID-19 pandemic. The digital divide refers to the uneven distribution of technological access and skill across ages, where older people have less access and lower proficiency in using technologies than younger adults. The authors explored the increased negative effects that the digital divide is having on the older population during the COVID-19 pandemic, while highlighting the need for increased attention and resources to improve digital literacy in the elderly. Intriguingly, this technological chapter clarifies that one of the few positive effects of the pandemic has been the acceleration of the application of telemedicine and digital medical and health tools.

## The Impact of the Pandemic on Ongoing Research

In the first wave of the pandemic, many ongoing research activities with human participants were halted to reduce face-to-face contact between participants and research staff. Through an anonymous self-administered online survey, McGoohan et al. investigated the willingness of PD patients and carers to resume clinical research and their opinion on adaptations to trials in light of COVID-19. The majority of respondents were positive about the continuation of non-COVID-19 related research as long as certain safety measures were in place, including using personal protective equipment, and research staff having regular tests for COVID-19 and traveling by car rather than public transport. Almost all (94%) indicated a willingness to complete assessments virtually, but telephone calls were the preferred method for remote follow-up compared to video call or online surveys. Thirty-nine percent of participants said they would feel more comfortable taking part in research if they did not have to visit a clinical setting, 8% preferred clinic settings, and the remainder were happy with either option. Regular and supportive communication from the research team was seen as important for maintaining the psychological well-being of participants while taking part in trials.

## Discussion

Countries are now facing their 2nd and 3rd waves of COVID-19. Although vaccination programmes are ongoing globally, periodical restrictions to reduce the rates of SARS-CoV-2 infection are being implemented; thus, results found in the first wave may be useful for providing better insights for the future. As the papers in the current Research Topic show, the restrictions may adversely affect older individuals in different ways. Nevertheless, the original research papers presented here predominantly analyzed data from the first wave of the pandemic in 2020 and the findings must be interpreted in light of this.

Many authors highlighted rising psychological effects of COVID-19 and the consequent restrictive measures adopted worldwide in patients with and without neurocognitive disorders. The emergence of new neuropsychiatric symptoms and a rapid increase of pre-existing symptomatology were reported at different stages of cognitive impairment, from both patients and caregivers, together with an increased use of psychotropic drugs. Furthermore, patients with symptomatic cognitive impairment or subjective cognitive complaints showed increased concerns about faster cognitive decline, with social isolation and reporting of one or more psychological symptoms considered as determining factors. It will be important to identify whether the neuropsychiatric symptoms that were often seen in patients with cognitive impairment and dementia in the first wave of the pandemic still increased in subsequent waves or whether people found better coping strategies over time.

The rapid negative change in routine clinical activities for non-urgent medical conditions during the pandemic affected not only patients' care access and monitoring but also increased caregiver burden. Apathy, aberrant motor behavior, sleep disorders, and psychosis increased in dementia patients and contributed to an increase in caregiver burden. Nevertheless, a comprehensive family support intervention on caregivers of patients with dementia was reported to reduce caregiver burden even in negative periods such as the COVID-19 pandemic. Furthermore, telemedicine and improving digital health literacy, together with psychosocial and psychoeducational person-centered interventions, were proposed as effective alternatives to manage patients' and caregivers' care during the pandemic emergency. It is essential to assess how effective such strategies have been for older individuals and whether they are sustainable in the post-pandemic era. Importantly, patient preferences and health equity must be considered, especially in relation to the digital divide that affects the older population.

This Research Topic also highlighted how the pandemic affected healthier older persons, in terms of lifestyle factors, among others. The “Dementia prevention, intervention, and care: 2020” report of the Lancet Commission (4) highlighted 12 modifiable factors risk factors that are estimated to account for around 40% of worldwide dementias, which consequently could theoretically be prevented or delayed. It is imperative to investigate what effect the pandemic-related changes in health and lifestyle behaviors will have on the future prevalence of dementia disorders. Further, intervention strategies to increase healthy lifestyle behaviors and promote social and cognitive stimulation during the ongoing pandemic need to be evaluated to identify which interventions are more successful at achieving behavior change in the short- and medium-term.

Studies in this Research Topic repeatedly demonstrated that the effects of the pandemic were particularly marked in individuals who live alone (Di Santo et al.; Lehtisalo et al.; Novotný et al.; Robb et al.; etc). As we move forward, it is crucial that people who have a higher risk of negative outcomes such as these are targeted for interventions to help them during future phases of the pandemic. Further, cross-country comparisons are needed to assess how lifestyle and health behaviors differed globally during the pandemic, depending on the various public health measures. Collaborative research and data harmonization between different study groups may play an essential role. For example, the World-Wide FINGERS network (6), a global network of trials that aim to prevent dementia and cognitive decline through risk factor modification, launched the WW-FINGERS SARS-CoV-2 Survey in multiple countries, to explore how the pandemic has affected risk factors for dementia, while accounting for country-specific strategies to contain the spread of the infection.

The progression of the pandemic is still unclear; we need to await long-term evidence concerning how long immunity persists after vaccination against COVID-19 and whether this differs between individuals according to individual characteristics (e.g., age, sex, ethnicity, etc). There is also uncertainty about SARS-CoV-2 Variants of Concern, and whether these may undermine current public health and vaccination strategies. Further, access to vaccination has not been equal for all countries; due to issues in production and supply, some low- and middle-income countries might have lower vaccination coverage than higher-income settings. Given all these uncertainties, it is likely that countries around the globe will need to periodically impose infection control measures to protect the population from COVID-19 and reduce the burden on healthcare systems. Thus, healthcare services need to plan strategies to deal with the emerging needs of older persons, patients with cognitive impairment and dementia, and those with psychological and neuropsychiatric symptoms. Initiatives need to deal with the screening, treatment, and monitoring of such symptoms during the ongoing pandemic as well as identifying strategies to deal with the rapid progression of cognitive and behavioral symptoms faced many individuals with pre-existing cognitive impairment, whose care has been significantly disrupted during the pandemic.

## Author Contributions

KP, NB, and GS wrote the editorial. KP and GS conceived the editorial and supervised the work. All authors read, performed critical revision, and approved the final version of the editorial.

## Funding

The Italian Ministry of Health (RC20-21/A) supported the present editorial.

## Conflict of Interest

The authors declare that the research was conducted in the absence of any commercial or financial relationships that could be construed as a potential conflict of interest.

## Publisher's Note

All claims expressed in this article are solely those of the authors and do not necessarily represent those of their affiliated organizations, or those of the publisher, the editors and the reviewers. Any product that may be evaluated in this article, or claim that may be made by its manufacturer, is not guaranteed or endorsed by the publisher.
